# Complementary Therapies of Diabetic Peripheral Neuropathy and Intermittent Claudication

**DOI:** 10.3390/healthcare13212676

**Published:** 2025-10-23

**Authors:** Shu Yan Ng

**Affiliations:** Pedorthic Technology Ltd., Rm 1101, 11/fl, Methodist House, 36 Hennessy Road, Wanchai, Hong Kong; ngshuyanhcc@gmail.com

**Keywords:** external counterpulsation, diabetic peripheral neuropathy, peripheral artery disease, intermittent claudication, diabetic feet, whole-food plant-based diet

## Abstract

Epidemiological studies have shown that the prevalence of diabetic peripheral neuropathy (DPN) will increase. Currently, DPN is treated medically. In many instances, the outcome is less than satisfactory, and the treatment is associated with side effects. We report a case of severe DPN and peripheral artery disease that are refractory to medical treatment. The patient was treated by external counterpulsation (ECP), electrical neuromuscular stimulation, a footbath in CO_2_-enriched water, and hydrogen inhalation, all of which are considered off-label. The patients also took L α-lipoic acid and vitamin B12 and were advised on lifestyle modification. The combination of treatments significantly improved the patient’s pain and claudication distance, increasing it from 16 m to 300–400 m. Additionally, as a result of the decrease in nocturnal pain, the patient experienced restful sleep. The reasons for the improvement in subjective symptoms are unclear, as changes in objective vascular and neurological measurements were inconsistent with the subjective improvement. This dissociation highlights the need for further research. Given the symptomatic relief observed, however, such alternative therapies could be considered on a case-by-case basis for patients with DPN who have limited treatment options.

## 1. Introduction

Diabetic peripheral neuropathy (DPN) is a common complication of type 2 diabetes mellitus (T2DM). Given the anticipated increase in the prevalence of T2DM, the prevalence of DPN is likely to surge, especially in low and middle-income countries [1]. A recent study in China showed that the prevalence of DPN in T2DM patients is as high as 67.6%, with 33.0% and 19.3% presenting with moderate and severe DPN, respectively [1]. The prevalence is significantly higher in females as compared to males and increases with age and the duration of diabetes mellitus [1].

The symptoms of DPN vary, depending on whether the condition affects the small nerve fibres, the large myelinated nerve fibres, or the peripheral arteries singly or in combination. DPN typically affects the small or C-fibres of the lower extremities first before the large myelinated nerve fibres [2], as small nerve fibres are unmyelinated and are more susceptible to mitochondrial dysfunction secondary to hyperglycemia. Since C-fibres are involved in pain and temperature sensation, early DPN typically presents with early-onset pain, characterized by burning or prickling sensations, which are generally worse in the evening and can disturb sleep [3]. The symptoms affect the patient’s quality of life and lead to polypharmacy, comorbidity, and socioeconomic consequences [3]. With the progression of the condition and degeneration of the large myelinated fibres, patients will experience leg numbness, tingling without pain, loss of vibration sensation, and loss of protective sensation in the feet [3]. Reduced proprioception may lead to poor balance and an unsteady gait, and increase the risk of falls [2,4]. Notably, patients with DPN tend to fall sideways, rather than forward or backward, due to a decrease in lateral stability resulting from sensory loss, muscle weakness, and poor proprioception. Sideways falls are associated with a risk of major fractures [5]. Some patients may present with insensate and numb feet, as if walking on thick socks [4]. A subset of patients, up to half of them, are asymptomatic or are reluctant to report their symptoms [6]. Clinicians should be aware that some patients who initially have neuropathic symptoms may become asymptomatic later in the course of the disease, as there is a severe loss of sensory nerves in all types of nerves [4].

The symptoms generally progress in a distal-to-proximal direction symmetrically, affecting both feet and hands in a sock-like distribution [7].

Typical medical treatment of DPN involves managing pain, controlling blood glucose levels, and providing proper foot care. Standard medical pain treatment involves anticonvulsants, opioids, selective norepinephrine and serotonin reuptake inhibitors (SNRI), and antidepressants [6]. They specifically target directly or indirectly to reduce pain, but they are associated with serious side effects or have addictive properties [6]. Additionally, they fail to address the underlying causes of the condition. Glycemic control alone, similarly, has not been found to effectively reduce DPN symptoms [8], as other factors such as neuronal ischemia [9], mitochondrial bioenergetics failure [10], accumulation of reactive oxygen species (ROS) [11], and inflammation [12] are involved in the pathogenesis of the condition.

Apart from standard medical treatment, some alternative or complementary therapies have been investigated for the treatment of DPN. These include the external counterpulsation (ECP) [13], electrical neuromuscular stimulation [14], carboxytherapy [15], hydrogen gas inhalation [16], and nutraceuticals [17,18]. They address the different aspects of the disease process. ECP has been reported to significantly improve the claudication distance in a patient suffering from DPN with intermittent claudication [13]. Calf-muscle electrostimulation has also been reported to enhance the claudication distance [14] substantially. The mean claudication distance of patients, with a mean age of 71 years, increased by 137 m from a baseline distance of 333 m after 91 days of stimulation [14]. Carboxytherapy has also been shown to significantly improve vibration, monofilament sensation, and temperature of the hallux in diabetic patients with a unilateral chronic wound [15]. Inhalation of hydrogen gas has been shown to exhibit potent antioxidant and anti-inflammatory properties [16].

Lifestyle modifications have also been found to positively impact DPN, stabilizing and improving it in over 80% of cases [19,20,21].

We present a case of DPN in an elderly patient with critical lower limb ischemia and diabetic foot lesions, whose condition was refractory to medical treatment and angioplasty. This report describes the application of a combination of alternative therapies after informed consent was obtained from the patient, who was experiencing increasing pain and diminishing walking distance.

## 2. Case Presentation

### 2.1. History

The male patient, aged 81, weighed 63 kg, was 152 cm high, and had a body mass index (BMI) of 27.3. He complained of pins and needles in the soles and pain in both calves when first seen in February 2025. He could only walk for around 16 m; his gait was unsteady, and he feared tripping, requiring a cane for ambulation. The patient graded the pain as 8 out of 10.

He had a long history of diabetes, hypertension, and hyperlipidemia. The T2DM has been present for 26 years and was treated by insulin injection (Ryzodeg 70/30 Flextouch). He was advised to inject 29 units before breakfast and 13 units before dinner daily. Other medications included gliclazide, empagliflozin, linagliptin, aspirin, famotidine, amlodipine, atenolol, doxazosin gits, losartan, atorvastatin, and mecobalamin (Table 1) to manage hyperglycemia, elevated cholesterol, and blood pressure.

In August 2024, he had an angioplasty of the right femoral artery, which was stenosed by more than 80%. Before the surgery, he could walk continuously for around 2000 m, with a VAS pain scale of 6/10. One week post-angioplasty, the pain dropped to 5/10, but worsened to 8/10 two months after the intervention, when he could only walk for around 16 m.

He smoked 20 cigarettes daily for more than 40 years. After the angioplasty, he reduced the number of cigarettes smoked to an average of four a day, but found it difficult to quit.

The patient sought alternative therapies as his condition did not improve with the standard medical treatments, knowing well that the treatments were off-label for DPN. Before the initiation of therapy, the patient was informed of the risks associated with deferring the revascularization proposed by the cardiologist. Also, he was told that treatment will be stopped if the pain increases, a wound develops, or dry gangrene expands in size. Given the severe pain that affected the quality of life of the patient and that the follow-up consultation by a cardiologist was three months away, the patient decided to try the combination therapies.

### 2.2. Examination

Upon presentation, the patient was assessed using the Flow 7 Device (LD Technology, Miami, FL, USA) for ankle-brachial pressure index and a sudomotor reflex response. We did not order any laboratory or ultrasound imaging tests, as these were performed in the hospital at no charge, and the patient was reluctant to have the tests repeated. Since the patient could only access his laboratory data, we therefore had only his laboratory findings. Ankle-brachial index (ABI) and sudomotor reflex response measurements at baseline on 25 January 2025 showed that the arteries around the ankles were incompressible with severely reduced toe brachial index (TBI) and a markedly reduced sudomotor reflex response value (Table 2), suggesting the presence of PAD and C-fibre dysfunction comorbidity.

The protective sensation of the feet was tested by the Semmes-Weinstein 5.07 monofilament (Happy Walking, Hong Kong) on 10 sites on each foot and was found intact.

**Figure 1:**
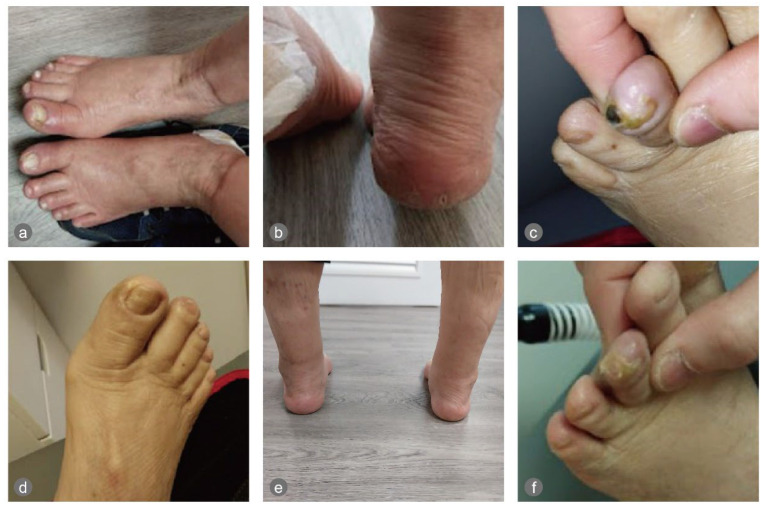
The feet pre- and post-intervention: (**a**) the onycholysis of the right hallux, (**b**) the cracked heels, and (**c**) small gangrene on the left third toe on 20 March 2025; (**d**–**f**) pictures of the feet post-intervention. In (**f**), it can be seen that the gangrene sloughed off (on 7 April 2025).

The physical examination revealed onycholysis of the right medial hallux and dry, painful, cracked heels on both feet. Signs of excoriations, erythema, and crusted lesions were also observed on the calves and legs, likely due to scratching secondary to dry skin and itchiness (Figure 1).

### 2.3. Intervention

The long history of T2DM, the reduced sudomotor reflex response, the loss of protective sensation, and the reduced TBI suggested that the patient had DPN. As previously indicated, he was treated by ECP, calf-muscle electrical neuromuscular therapy, CO_2_ therapy, and hydrogen therapy. Additionally, he was advised to modify his diet to a low-fat, whole-plant diet.

As he had critical ischemia of the legs, the ECP treatment was conducted with extra care. We raised the patient’s heels slightly by placing towels proximal to the heels, as his heels were painful when simply resting on the couch. Also, we covered his trunk and thighs with a blanket, leaving the feet uncovered, as the onycholysis of his right hallux could not tolerate the weight of a blanket. Treatment pressure was set at a maximum of 4 PSI, with an average of 2.4 PSI. The patient was continuously monitored. During the first few sessions, the treatment had to be stopped after around 7 min, as the patient complained of numbness and cramps in both legs. The cramps were relieved with magnesium oil spray, after which the treatment was resumed. It was stopped again when the patient experienced leg cramps. In the first few treatments, the treatment was fragmented, but added up to 50 min. The frequency of cramps and numbness gradually decreased with the number of ECP treatments, and the time it took to feel numbness and aches in the calves during therapy increased from 7 min to around 20 min. After a few therapy sessions, the patient was able to sleep through the entire hour of treatment.

Given the severity of his PAD, the patient was also prescribed a neuromuscular stimulator, which stimulated arterial blood flow to the lower extremities The key parameters include a biphasic, rectangular waveform, a pulse duration of 25–240 microseconds, a frequency that can induce 2–120 contractions per minute, a low energy output of <5 microcoulombs per pulse, and a peak voltage of <50 V. They are not adjustable; the only adjustable parameter is the current output. The patient was instructed to apply each electrode to each calf and set the output current to a level that was maximally tolerable. Treatment was once or twice a day.

The patient was also advised to soak his feet in the CO_2_-enriched water with a pH of 7.21 (Table 2) for 20 min every evening at home. The water was prepared by dissolving a 16 g carbon dioxide spa tablet in a litre of water at 37–38 °C.

Further, he used a Naqi cream to treat the dry skin and the hyperoil gel to manage the onycholysis of the right hallux. The hyperoil was applied twice daily from February to April 2025. He was advised to take B12, Lα-lipoic acid, vitamin K2 with D3, CoQ10, and nattokinase (2000 FU × 2) supplements.

### 2.4. Outcome and Follow-Up

On the night of the first ECP treatment, the patient slept for 10 h. The treatments were uneventful. The cracked heels healed in around 7 days, and the onycholysis healed in about two months. The necrotic tissue of a small gangrene discovered on the tip of the left third toe on 20 March 2025 sloughed off on 7 April 2025 (Figure 1), when he could start wearing diabetic shoes instead of sandals.

**Figure 2:**
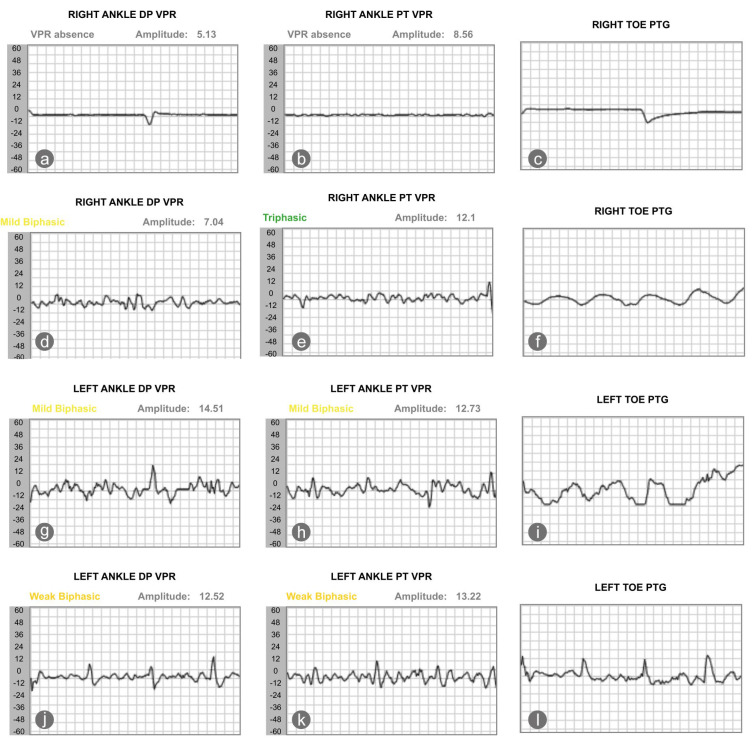
The changes in pulse wave, as recorded by the photoplethysmography, showed that changes of blood volume in the microvascular bed of tissue of the right (**a**–**c**) and left (**g**–**i**) foot at baseline and after intervention (**d**–**f**) for the right and left foot (**j**–**l**). DP stands for dorsalis pedis; VPR stands for volume photoplethysmography recording; PT stands for posterior tibialis artery, and PTG indicates photoplethysmography.

On 1 June 2025, his vascular status and sudomotor reflex response were reassessed. Results showed that the sudomotor reflex response, the nitric oxide peak, and the patient’s ABI slightly worsened. Yet, the perfusion of the right toe improved; the TBI of the right second toe increased from 0.15 to 0.36 (Table 2). We also saw an improvement in the blood volume of the dorsalis pedis and posterior tibialis artery, as recorded by photoplethysmography. The volume plethysmography records (VPR) of the right ankle dorsalis pedis and posterior tibialis arteries were minimal in January 2025. In May 2025, the VPR of the right dorsalis pedis and posterior tibialis arteries improved, becoming mildly biphasic and triphasic, respectively. The TBI of the left foot, in contrast, decreased from 0.58 to 0.31, with the VPR of the left dorsalis pedis and posterior tibialis artery worsened from mildly biphasic to weakly biphasic (Figure 2).

The VAS was reduced to 3/10 after 60 ECP treatments, and he was able to walk 300–400 m, compared to 16 m before the intervention.

As of 16 June 2025, he had reduced the insulin injection from 29 units before breakfast to 27 units and from 13 units to 12 units before dinner.

Interestingly, the patient reported that around one month after drinking hydrogen-enriched water, the foamy nature of his urine significantly decreased, allowing him to notice the color of his urine for the first time in many years. We are uncertain of the clinical significance, as it was not confirmed by laboratory testing. The patient declined private urinalysis, preferring to wait for his scheduled hospital appointment.

On 2 April 2025, his cardiologist performed an ultrasonic examination of the arteries in the lower extremities and suggested that he have angioplasty and possibly bypass graft surgery of the arteries of both legs. However, the cardiologist cautioned him of the risk of contrast-induced nephropathy and the need for haemodialysis, given that his eGFR was only 37 mL/min/1.73 m^2^. The patient was scheduled for further consultation with the cardiologist three months later. The patient, fully understanding the potential risks and benefits, decided to continue with the combination therapies as symptoms improved, and he had three months to decide.

Concerned about the possibility of future angioplasty, the patient attempted to adopt a WFPBD and reduced his intake of saturated fat, trans fat, and animal products. The lifestyle modification, however, was challenging for him. On the one hand, his wife had mild cognitive impairment, and he had to prepare the food himself. Additionally, he was unable to tolerate many legumes and beans, as they triggered gouty attacks. So, basically, he only reduced his meat intake.

He did not engage in any meaningful exercises, despite being counselled on the importance of exercise in managing T2DM and DPN [22,23].

## 3. Discussion

The decision to pursue alternative therapies in this patient with advanced PAD and polyneuropathy required careful ethical consideration. The patient’s marked deterioration following a previous angioplasty was the primary reason for his reluctance to undergo further immediate revascularization. The combination therapy was pursued as an interim measure during the wait for his next cardiology consultation, with the patient’s full informed consent regarding their experimental nature and the potential risks of delaying standard interventions.

The different therapies employed have been studied for their impact on PAD and DPN.

ECP may benefit PAD and improve walking distance. Braith et al. (2010) examined patients with angina undergoing ECP treatment and showed a 30% increase in flow-mediated dilation in the femoral artery [24]. Werner et al. (2007) [25] reported that when the cuffs inflated and deflated during the treatment, the average blood flow volume of the posterior tibial artery decreased by one-third. However, one hour post-procedure, blood flow increased to 133% of the baseline value [25]. Similar results were reported by Zhang et al. (2024), who showed that ECP treatment improved blood flow volume and velocity in the lower extremity [26]. The increase in vascular shear stress during the intervention may enhance nitric oxide release [27], thereby contributing to improved blood perfusion in the lower extremities and an increase in walking distance [26]. Ng (2025) described a case of DPN with intermittent claudication responding to ECP treatment. The patient significantly increased his claudication distance [13].

Additionally, studies have shown that patients with PAD receiving ECP treatment did not experience any adverse effects [28]. Thakkar et al. (2010) [28], using the International EECP Patient Registry (IEPR), found that 23% of the patients with angina undergoing ECP treatment had concomitant PAD. They found that the short and long-term improvements following the ECP treatment did not differ between the PAD group and the non-PAD group [28], supporting the use of ECP in PAD. More importantly, perhaps, ECP promotes angiogenesis [29], thereby improving vascular perfusion over a more extended period.

CO_2_ therapy has been used in the management of diabetic-related feet in Europe for over 80 years [30]. Many studies have demonstrated that carboxytherapy effectively alleviates symptoms in patients with DPN [15,31]. A prospective, randomized, double-blind study [15] demonstrated that transcutaneous carbon dioxide therapy significantly improved vibration, monofilament sensation, and temperature of the hallux compared to air. Bulum et al. (2025) similarly showed that carbon dioxide therapy significantly restored protective sensations in patients with diabetic-related foot ulcers [32]. Dogaru et al. (2025) demonstrated that addition of carbon dioxide mineral bath to comprehensive medical rehabilitation program which includes physical exercises, physiotherapy, unipolar galvanic baths for the lower limbs for 10 min/day, transcutaneous electrical nerve stimulation for neuropathic pain control for 10 min/day and outdoor therapy for 30 min/day significantly reduced pain of DPN patients as compared to those who received the rehabilitation program only [31]. The improvement persisted for three months post-treatment. The outcome is attributed to the fact that carbon dioxide dilates blood vessels and induces neoangiogenesis [33,34,35]. These effects also explain why carboxytherapy has been used as an adjunctive treatment for critical limb ischemia [36] and diabetic ulcers [32,37,38].

Electrical neuromuscular stimulation was also prescribed, as studies have shown that it significantly improves claudication distance [14]. The therapy involves stimulating the calf muscles for an hour daily, consecutively for 12 weeks, using a program with varying frequencies ranging from 1–250 Hz. The approach significantly improves vascular perfusion, increases claudication distance in diabetic patients with PAD, and improves ABI [14]. The absolute claudication distance increased from a mean of 333 m to 470 m. Also, the electrostimulation enhances collateral artery growth [14]. As the results were so encouraging, the authors proposed using electrostimulation as a therapy for the high-risk group of patients with diabetes mellitus, bilateral limb ischemia, and vascular claudication [14].

Patients were also advised to inhale hydrogen, as it is a powerful antioxidant and anti-inflammatory agent, given that DPN is associated with oxidative stress and inflammation. Studies on DPN primarily focused on animals, with human studies being relatively few.

Studies [39,40,41,42] have shown that hydrogen administration improves motor nerve conduction velocity in rats with DPN and the functional impairment of neuropathic hyperalgesia in diabetic rats [40]. Han et al. (2023) demonstrated that hydrogen administration significantly increased the density and average sectional area of myelinated nerves, which were previously broken, damaged, or missed in diabetic rats, suggesting that hydrogen may reduce nerve damage caused by hyperglycemia and oxidative stress [39]. Additionally, animal studies have shown that hydrogen improves motor nerve conduction velocity [39,40,41,42]. Wang et al. (2022) demonstrated that hydrogen-rich saline alleviated the symptoms and functional impairment of neuropathic hyperalgesia in diabetic rats, with the effect correlated to a reduction in pro-inflammatory cytokine levels [40]. Both the thermal withdrawal latency and mechanical withdrawal threshold were reduced [40]. The combination of treatments significantly improved balance and claudication distance (Table 2), alleviated nocturnal leg pain and paresthesia, and enhanced sleep quality. The improvement of subjective symptoms is unlikely to be due to the standard medical treatment, as it had been employed well before the intervention. Likewise, the changes might not be due to the hydrogen inhalation or WFPBD, as the patient inhaled hydrogen after the initial 35 sessions of ECP. Additionally, he did not strictly adhere to the WFPBD. Alpha-lipoic acid and vitamin B12 may play a role in improving symptoms [17,43]. We are unsure if a reduction in the number of cigarettes smoked had any impact. We thus are unable to attribute the benefits to any specific intervention. Given this, our objective was not to investigate which specific therapy can help alleviate DPN symptoms, but whether combination therapies can help alleviate DPN symptoms in patients with limited options. As different interventions have been employed in the management of the case, it is challenging to isolate the effects of each one. Yet, the timelines of the intervention provide some clues to the impact of the multimodal therapies. Studies showed that oral supplementation of vitamin B12 and α-lipoic acid took a few weeks to bring about symptomatic relief [44]. Also, the patient modified his diet after 35 sessions of ECP treatment, approximately two and a half months into the treatment. As the improvement in symptoms occurred during the first week of multimodal therapies, the off-label combination therapies likely played a part in improving the condition.

Yet, the duration of the improvement cannot currently be determined. The improvement may be transient. We shall, however, follow up on the case closely and report the outcome in the future.

What is intriguing is that the patient’s subjective improvement contrasts with most objective findings. The only vascular parameter that aligned with clinical improvement was the right TBI, indicating that therapeutic interventions may have successfully enhanced perfusion to that limb. Conversely, the bilateral elevation in ABI, the decline in left TBI, and the bilateral reduction in sudomotor responses all indicate ongoing disease progression. This pattern suggests that while therapy provided localized vascular benefit and symptomatic relief, it was insufficient to counteract the advancement of peripheral arterial disease in the left foot or the progression of diabetic polyneuropathy.

It is possible that the reduction in pain and improvement in walking distance reflected that the feet were becoming insensate. Yet, clinically, we found the possibility unlikely. The improvement of symptoms began within a few days of the intervention, and the patient still had some protective sensation. However, we cannot disregard the potential role of placebo effects or nonspecific effects of the multimodal therapies in the patient’s improvement.

The findings underscore the potential of the combination therapies to significantly improve patient symptoms. However, it is important to note that these therapies did not improve the objective findings. The dissociation underscores the crucial need for further studies to fully understand whether the intervention can enhance vascular perfusion and small fibre dysfunction over a more extended period, given the slow regeneration of C-fibres [45].

This study has several limitations inherent to its design as a single-case report. The patient’s primary management occurred in a hospital setting, which limited our access to certain data, including the original arterial ultrasound imaging, preventing a comprehensive comparison of vascular structures at baseline and follow-up. Also, our initial assessment was not exhaustive; we did not include the 6 min walk test and the neuropathy disability score (NDS). Furthermore, adherence to the home-based interventions (neuromuscular stimulation, foot baths, and the WFPBD) was based on patient recall rather than monitored quantification. The absence of a control group also means that the observed improvements cannot be definitively attributed to the interventions. Future controlled studies with rigorous monitoring of adherence and comprehensive objective measurements are warranted to validate whether this combination of therapies can effectively improve claudication and neuropathic symptoms in patients with treatment-refractory DPN.

## 4. Conclusions

In conclusion, this case report suggests that a combination of off-label therapies may improve symptoms in patients with DPN who are refractory to standard care. While the symptomatic results are promising, they must be interpreted with caution due to the study’s limitations, including its single-case report nature and the use of multiple simultaneous interventions. Further research is required to validate these findings and to determine the objective basis of the outcome. Until more data is available, clinicians may consider these approaches on a case-by-case basis, ensuring thorough discussion of the potential risks and unknown benefits with their patients.

## Figures and Tables

**Table 1:** The duration of T2DM, the complaints, and the patient’s medications.

	Sex/Age	M/81
	Years of T2DM	26
Medications	Antihypertensives	Amlodipine, atenolol, doxazosin gits, losartan
	Statins	Atorvastatin
	Anti-glycemic	Gliclazide, empagliflozin, linagliptin, insulin injection
	Pain Killers	NA
	Other Medications	Famotidine, mecobalamin
Complaint		Unsteady gait; unable to walk for more than 16 m

**Table 2:** The results of complementary therapies and standard medical treatments at 4–6 months of intervention. It is worth noting that the outcome was not all measured at the same specified time; instead, it occurred between 4 and 6 months after intervention, as laboratory and quantitative nerve tests were conducted at different time frames. PEA stands for palmitoylethanolamide. ABI stands for ankle-brachial index; TBI for toe-brachial index, R stands for right, and L stands for left.

Complementary Intervention	ECP	First 35 therapies (5/weekly, for 7 wks)
		Thereafter, twice a week
	Veinoplus arterial	1–2 h/day
	CO_2_-enriched water (pH7.21) footbath	1/day; 20 min each time
	Hydrogen gas inhalation	3–4 h/day (commenced after the first 35 ECP tx)
Nutraceuticals	Prescribed by medical physicians	CoQ10, vitamin C, vitamin B complex
	Self-prescription	NA
	Our advice	PEA (100 mg bid), lipoic acid (300 mg qd), vitamin K2 and D3 (K2:100 mcg, D3:10 mcg bid), nattokinase (2000 FU tid)
Lifestyle	Quit Smoking	From 20 to 4 cigarettes/day
	Low-fat WFPBD	Inconsistent (after 35 ECP tx); cannot tolerate legumes.
	Exercises	Walk 300–400 m/daily at present.
Outcome after Intervention	Nocturnal paresthesia and pain	From 8/10 to 2/10
	Sleep Quality	No more insomnia.
	Gait	More steady; markedly improved.
	Walking distance	Increased from 16 m to 300–400 m
	Withings Nerve Score	Improved from 44 to 58 microSiemens (μS).
	ABI	R: 1.28 to 1.4; L: 1.25 to 1.38
	TBI	R: 0.15 to 0.36; L: 0.58 to 0.31
	Sudomotor Reflex Response (mv)	R: 550 to 525; L: 550 to 448
	eGFR (mL/min/1.73 m^2^)	From 37 to 38

## Data Availability

As the dataset contains detailed personal identifiers, the data presented in this study are available upon request from the corresponding author to protect patient confidentiality.
